# Optimising fluorescein diacetate sputum smear microscopy for assessing patients with pulmonary tuberculosis

**DOI:** 10.1371/journal.pone.0214131

**Published:** 2019-04-30

**Authors:** Sumona Datta, Keren Alvarado, Robert H. Gilman, Teresa Valencia, Christian Aparicio, Eric S. Ramos, Rosario Montoya, Carlton A. Evans

**Affiliations:** 1 Infectious Diseases & Immunity, Wellcome Trust Centre for Global Health Research, Imperial College London, United Kingdom; 2 IFHAD: Innovation For Health And Development, Laboratory of research and development, Universidad Peruana Cayetano Heredia, Lima, Peru; 3 Innovacion Por la Salud Y el Desarollo (IPSYD), Asociación Benéfica Prisma, Lima, Peru; 4 Department of International Health, Johns Hopkins Bloomberg School of Public Health, Baltimore, MD, United States of America; Jamia Hamdard, INDIA

## Abstract

**Background:**

Assessing *Mycobacterium tuberculosis* (TB) viability by fluorescein diacetate (FDA) microscopy can predict TB culture results, treatment response and infectiousness. However, diverse methods have been published. We aimed to optimise FDA microscopy, minimising sputum processing, biohazard and complexity for use in resource-constrained settings.

**Methods and results:**

Optimization: Patients with smear-positive pulmonary TB before treatment and healthy control participants provided sputa. These were divided into equal aliquots that were tested directly or after NaOH centrifuge-decontamination. Each aliquot was cultured and used to prepare slides (n = 80). FDA microscopy used: 1 or 3 drops of sputum; with/out acid-alcohol wash; with/out phenol sterilization; with 0/30/60 seconds KMnO_4_ quenching. Control samples all had negative culture and microscopy results. FDA microscopy had higher sensitivity when performed directly (without centrifuge-decontamination) on 1 drop of sputum (P<0.001), because 3 drops obscured microscopy. Acid-alcohol wash and KMnO_4_ quenching made bacilli easier to identity (P = 0.005). Phenol sterilization did not impair microscopy (P>0.1). Validation: The 2 protocols that performed best in the optimization experiments were reassessed operationally by comparing duplicate slides (n = 412) stained with KMnO_4_ quenching for 30 versus 60 seconds. FDA microscopy results were similar (P = 0.4) and highly reproducible, with 97% of counts agreeing within +/-1 logarithm. Storage: Smear microscopy slides and aliquots of the sputum from which they were made were stored for 4 weeks. Twice-weekly, paired slides (n = 80) were stained with freshly prepared versus stored FDA and read quantitatively. Storing sputum, microscopy slides or FDA solution at 4°C or room temperature had no effect on FDA microscopy results (all P>0.2). Cost: Material costs for each slide tested by FDA microscopy using reagents purchased locally were USD $0.05 and required the same equipment, time and skills as auramine acid-fast microscopy.

**Conclusions:**

We recommend a simple, bio-secure protocol for FDA microscopy that provides sensitive and repeatable results without requiring centrifugation.

## Introduction

In 2017, more than 6.7 million cases were notified as having pulmonary tuberculosis (TB), but 44% of them did not have microbiological confirmation of diagnosis or cure [1]. The reasons for this are multifactorial, including limited access to appropriate-technology tests, poor test performance and poor quality of diagnostic samples [2,3]. Culture is the gold-standard diagnostic method for TB as it is able to identify paucibacillary disease and definitively determine drug susceptibility. Unlike conventional acid-fast microscopy and PCR methods, culture also discriminates between live and dead/non-replicating bacilli. Therefore, culture is used to confirm TB disease, drug resistance, and monitor treatment response, including in anti-tuberculous drug trials. However, as culture is technically challenging and biohazardous and most settings with a high TB burden have limited resources, the majority of TB care is guided by microscopy.

Fluorescein diacetate (FDA) is a vital stain that generally causes viable cells to fluoresce because non-specific esterase in the cytoplasm of metabolically-active cells must be present to hydrolyse the stain to a fluorescent form [4]. FDA was first demonstrated to indicate esterase activity in mammalian cells by Rotmann and Papermaster, followed by Medzon and Brady’s study demonstrating its application in numerous bacteria in 1969 [5,6]. Since then it has been used in various fields of biology to assess cell viability, especially for organisms that are difficult to culture. For example, FDA has been used to monitor treatment response in patients receiving therapy for leprosy [4,7]. However, FDA microscopy uses fluorescence that until recently required expensive mercury or halogen light sources, which previously limited feasibility for routine use in clinical settings.

In the 1980s, FDA was demonstrated to stain *Mycobacterium tuberculosis*, and was subsequently shown with clinical specimens to predict in 1 hour the results of TB culture that would only be available weeks later [8–10]. In 2006, a group in Bangladesh reported that sputum smear microscopy with FDA could be used to identify culture-confirmed treatment failure in patients who had positive conventional smear microscopy results after 2 months of first-line treatment [11]. Consequently, they implemented the use of sputum FDA microscopy for patients suspected of having a failed Category I or II treatment in 4 regional laboratories. This predicted multi-drug resistant (MDR)-TB with 93% accuracy [12] and led to 23% more patients switching to appropriate second-line treatment earlier than with the previous use of reference laboratory culture results [12]. Concurrently in Peru, the number of fluorescing bacilli seen with FDA microscopy during the first 9 days of first-line anti-TB therapy rapidly predicted treatment response and the presence of MDR-TB [13,14]. Furthermore, FDA microscopy results for sputum samples prior to starting treatment identified the most infectious patients [15].

Affordable light-emitting diode (LED) microscopes are now widely available and the World Health Organization encourages their use with auramine staining for sputum smear microscopy in place of conventional light microscopy with Ziehl-Neelsen staining [16]. The widespread and increasing availability of fluorescence microscopes may allow FDA microscopy to have greater clinical applicability.

Review of the published protocols for FDA microscopy for TB identifies diverse methods used, as shown in Table 1 [8,9,12,13]. However, there is no published evidence to guide which of these protocols should be used. Variability in methods may lead to misleading interpretations due to false-positive or false-negative results [17]. Additionally, some protocols involve sputum processing with centrifuge decontamination, which is a barrier to implementation in most microscopy centres because centrifugation may be biohazardous and centrifuges with sealed rotors suitable for use for TB diagnosis are expensive and have limited availability.

**Table 1:** Comparison of FDA protocols published in English for staining *Mycobacterium tuberculosis*. Note, NS = not specified, mins = minutes, CPC = cetylpyridinium chloride, and NAOH-NALC = sodium hydroxide and N-acetyl cysteine.

	Jarnigin [8]	Datta (13)	Salim [11]	Schramm [17]	Van Deun [12]
**Stock solution **					
Dissolvent	acetone	acetone	NS	acetone	NS
FDA concentration (mg/ml)	5	5	5	25	0.5
Storage temperature (°c)	4	-20	-20	-20	-20
Maximum storage (days)	56	730	730	730	730
**Working solution**					
Dissolvent	Dubos albumin broth	40% acetone	acetone	acetone	acetone
FDA concentration (mg/ml)	0.5	0.02	0.05	0.5	0.02
Storage temperature (°c)	NS	4	-20	NS	-20
Surfactant added for storage	Tween 80	-	Tween 80	Tween 80	Tween 80
Maximum storage (days)	NS	1	7	NS	7
**Sputum sample processing**					
Preservative for transport	used culture suspension	-	CPC	-	-
Decontamination		4% NAOH-NALC	-	4% NAOH-NALC	-
Centrifugation		3000g	-	NS	-
**Staining**					
Staining area (cm^2^)	1	1	NS	1	NS
Sample volume (drops)	1	3	NS	3	NS
Slide fixation	flame	serum albumin and flame	-	-	-
FDA application	filter paper	filter paper	NS	filter paper	NS
FDA incubation time (mins)	30	20	30	30	30
Acid-alcohol step	-	-	1% for 1–2 mins	1% for 3 mins	0.5% for 3 mins
Sterilization step	-	-	5% phenol for 10 mins	5% phenol for 10 mins	5% phenol for 10 mins
Quenching step	-	-	-	-	0.5% KMnO4 for 1 min
Coverslip	yes with glycerol	-	-	-	-
**Reading **					
Microscope description	BD-12 primary filter	Nikon	Mercury vapor system	Olympus CX21 LED	FluoroLED 450nm
Magnification	450	1000 (oil)	1000 (oil)	1000 (oil)	200

We therefore aimed to:

- select the optimum FDA microscopy protocol that is simple and safe;
- refine the staining method;
- assess whether storage conditions of sputum or FDA working solutions affect results;
- and determine the cost of this protocol.

This has allowed us to propose a standard operating procedure for FDA microscopy that can simply and safely provide reproducible results in resource constrained clinical settings.

## Materials and methods

### Ethics

Approvals included Imperial College London and the Peruvian Ministry of Health DIRESA Callao. This research was done with the collaboration of the Peruvian national TB program.

### Setting

Patient sputum samples were collected from adults diagnosed with pulmonary TB in 15 community health centres in the peri-urban shantytowns of Callao, Peru. The study involved 3 phases: 1. optimisation study; 2. validation study; and 3. storage study. In both the optimisation study and the storage study, control samples were collected from asymptomatic, healthy individuals.

### Inclusion and exclusion criteria

Inclusion and exclusion criteria are detailed below for each experiment.

### Samples

Sputum samples were processed even if they appeared to be salivary. All sputum samples were collected and transported to the local, research laboratory at ambient temperature and then stored at 4°C and processed within 24-hours of arrival, usually within 72 hours of expectoration.

### Slides

All slides were prepared by smearing sputum over standard glass microscope slides that had been cleaned with 95% alcohol.

### Measurements

To increase operational relevance, we measured liquids including sputum as drops from disposable transfer pipettes, which we found to have an average volume of 40 μl.

### Slide preparation

Microscopy using a 100x objective to examine 100 high power fields assesses an area of 1–2 mm^2^ [18]. For clarity, we therefore report the volume of sputum smeared per mm^2^ of the area of the glass microscopy slide.

### Sputum smears

‘Thin smears’ were prepared at a typical density used for sputum smear microscopy by smearing 1 drop over a 2 cm^2^ area i.e. 0.2 μl/mm^2^. ‘Thick smears’ were made with a higher density than is usually used for sputum smear microscopy by smearing 3 drops over an area of 1 cm^2^ i.e. 1.2 μl/mm^2^, as previously used for FDA microscopy, see Table 1 [13,14,17].

### Fixation

All sputum smears were then heat fixed to the slide by passing each slide through a flame 3 times.

### Microscopy

All stained slides were dried, protected from light and for fluorescence microscopy were read with Zeiss (Heidenheim, Germany) iLED microscopes using the 100x objective with oil immersion without using microscopy cover slips.

### Blinding

All slides prepared in the same way were ‘shuffled’ before staining to ensure that the order in which slides were prepared did not influence the order in which they were processed, nor the protocol used. Microscopy was performed by multiple laboratory biologists and technicians who were always unaware of the clinical status of the patient, and the results of other tests.

### Optimisation study

#### Inclusion criteria

This initial optimisation study used sputum samples that were either from: selected patients who had already been determined to have a positive acid-fast microscopy result but had not yet received any TB therapy; or healthy negative control participants with neither symptoms nor suspicion of TB disease, during 6 May until 20 July 2015.

#### Exclusion criteria

Exclusion criteria were lack of informed written consent or inability to produce a sample.

#### Sputum processing

Reagents were obtained from Thermo Fisher Scientific (MA, USA) except where otherwise stated. Sputum samples were divided into aliquots that were: processed with centrifuge decontamination; or left unprocessed at room temperature to be directly smeared onto slides.

#### Centrifuge decontamination

Centrifuge decontamination was done as described [19]. Briefly, the 2 ml aliquot for decontamination was briefly vortexed with an equal volume of 2% sodium hydroxide containing 2.9% sodium citrate and 0.5% N-acetyl-L-cysteine. After 20 minutes at room temperature, excess phosphate buffered saline at pH 6.8 (Sigma-Aldrich, MO, USA) was added, and the mixture was centrifuged for 15 minutes at 3,000 gravities in a Thermo Fisher Scientific centrifuge with sealed rotors to increase biosafety for the laboratory personnel [19]. The pellet was re-suspended in phosphate buffered saline at pH 6.8 to a final volume of 2 ml.

Both decontaminated and unprocessed aliquots were then used for slide preparation and culture inoculation.

#### Solutions

A stock solution of 5 mg/ml FDA (Sigma-Aldrich, MO, USA) in acetone was stored at -20°C. A fresh working solution of 20 μg/ml FDA was prepared daily by dissolving 10 μl of stock solution in 2.5 ml of 40% acetone in phosphate buffered saline at pH 6.8. Standard acid-alcohol (AA) solution used in TB fluorescent microscopy was prepared by adding 0.5% hydrochloric acid to 96% ethanol [20]. Phenol solution was prepared by adding 5% phenol (Merck, NJ, USA) to distilled water. Potassium permanganate solution was prepared by dissolving 0.5% KMnO_4_ (Merck, NJ, USA) in distilled water [20].

#### Smears

A thick smear was prepared from the centrifuge-decontaminated aliquot and 2 thick smears and 5 thin smears were prepared from the direct aliquot. Aliquots of each sample were processed concurrently with all of the following FDA staining protocols, as described below, in Box 1 and in Table 2.

**Box 1. Protocols for fluorescein diacetate (FDA) microscopy in the optimisation experiment.**

**FDA staining protocol A (Centrifuge-thick)** was processed as published [13], by applying 3 drops of centrifuge-decontaminated sputum to a slide to make a smear of approximately 1 cm^2^.

**FDA staining protocol B (Direct-thick)**

1. Using cleaned slides, 3 drops of unprocessed sputum were smeared over an area of approximately 1 cm^2^. The slides were then dried and protected from ultraviolet light.
2. When slides were dry, they were passed over a flame 3 times.
3. A 1 cm^2^ square of Whatman grade 3 filter paper was placed on top of the smear, and 13–15 drops of freshly prepared FDA working solution at a concentration of 20 μg/ml FDA was applied to cover the filter paper.
4. Slides were then incubated for 30 minutes at 37°C.
5. After removing the slides from the incubator, excess liquid was tapped off.

**FDA staining protocol C (Direct-thick-AA)**

Same as protocol B, except with the following added steps:

6. Slides were then rinsed with distilled water.
7. Afterwards 0.5% acid-alcohol (AA) was flooded onto the slides and left for 3 minutes.
8. Slides were then rinsed with distilled water.

**FDA staining protocol D (Direct-thin-AA)**

1. Using cleaned slides, 1 drop of unprocessed sputum was smeared over an area of approximately 2 cm^2^. The slides were then dried and protected from ultraviolet light.
2. When slides were dry, they were passed over a flame 3 times.
3. 13–15 drops of freshly prepared FDA working solution at a concentration of 20 μg/ml FDA was applied to the sample to cover the smear.
4. Slides were then incubated for 30 minutes at 37°C.
5. After removing the slides from the incubator, excess liquid was tapped off.
6. Slides were then rinsed with distilled water.
7. Afterwards 0.5% AA was flooded onto the slides and left for 3 minutes.
8. Slides were then rinsed with distilled water.

**FDA staining protocol E (Direct-thin-AA-phenol)**

Same as protocol D, except with the following steps:

9. Phenol at 5% concentration was applied to slides and left for 10 minutes.
10. Slides were then rinsed with distilled water.

**FDA staining protocol F (Direct-thin-AA-phenol-KMnO_4_30s)**

Same as protocol E, except with the following added steps:

11. Potassium permanganate at a concentration of 0.5% was applied to slides and left for 30 seconds.
12. Slides were then rinsed with distilled water.

**FDA staining protocol G (Direct-thin-AA-phenol-KMnO_4_60s)**

Same as protocol E, except with the following added steps:

1. Potassium permanganate at a concentration of 0.5% was applied to slides and left for 60 seconds.
2. Slides were then rinsed with distilled water.

**Reading slides**

1. After staining, all the slides were left to dry in the dark.
2. Slides were then read within 4 hours of staining
3. Using the fluorescent light source and 100x objective with oil immersion on the Zeiss iLED microscope (Heidenheim, Germany), the number of bacilli visible in 100 fields was recorded.

**Table 2:** Optimisation study. Table demonstrating the different protocols of fluorescein diacetate (FDA) microscopy, the number samples, and quality assessment. Please see methods and Box 1 for full explanation of the protocols.

		FDA PROTOCOLS						
		A. Centrifuge-thick.	B. Direct-thick	C. Direct-thick-AA	D. Direct-thin-AA	E. Direct-thin-AA-phenol	F. Direct-thin-AA-phenol-KMnO_4_30s	G. Direct-thin-AA-phenol-KMnO_4_60s
		Datta (13)						Van Deun [12]
**Staining method**	Decontamination	2% NaOH	no	no	no	no	no	no
	Drops of Sputum (uL)	3 (120)	3 (120)	3 (120)	1 (40)	1 (40)	1 (40)	1 (40)
	20ug/ml FDA incubation (min)	20	20	20	20	30	30	30
	0.5% Acid-alcohol (min)	-	-	2	2	3	3	3
	5% phenol (min)	-	-	-	-	10	10	10
	KMnO4 (sec)	-	-	-	-	-	30	60
**General results**	Number of samples, including 2 negative controls for each technique	11	11	11	9	11	5	11
**Quality markers**	Background score, median (IQR)*	67 (33–100)	24 (0–33)	67 (33–67)	33 (33–67)	67 (33–67)	67 (67–67)	100 (100–100)
	Bacillary brightness score, median (IQR)*	40 (0–60)	60 (0–80)	60 (0–80)	60 (40–80)	80 (40–100)	60 (20–60)	40 (40–60)
	Bacillary identification score, median (IQR)*	33 (0–33)	33 (0–33)	33 (0–33)	33 (33–100)	66 (33–100)	100 (33–100)	100 (67–100)
	Easy to focus, % (n)	73% (8)	92% (10)	64% (7)	89% (8)	82% (9)	80% (4)	56% (6)
	Total quality score, median (IQR)**	200 (200–240)	206 (100–247)	213 (133–240)	240 (206–280)	260 (240–333)	327 (253–326)	273 (240–306)

Footnote.

* the score was made from Likert-type scales and transformed to a score out of 100, where 100 was the best and 0 was the worst.

** the total quality score was a sum of the score for background, brightness, identification and focus, therefore the maximum and best score that could be achieved was 400.The median here refers only to the patient samples.

#### FDA staining protocol A (Centrifuge-thick)

FDA staining protocol A (Centrifuge-thick) used thick smears from the centrifuge-decontaminated aliquot. FDA staining was done by covering the smear with a 1 cm^2^ square of Whatman grade 3 filter paper that was soaked with FDA working solution and incubated at 37°C for 20 minutes, after which the filter paper was discarded, excess FDA was tapped off the slide, and left to dry prior to microscopy. As shown in Table 1, we had used and published this protocol previously [13].

#### FDA staining protocol B (Direct-thick)

FDA staining protocol B (Direct-thick) assessed modifying protocol (A) only by using a thick smear of unprocessed instead of centrifuge-decontaminated sputum.

#### FDA staining protocol C (Direct-thick-AA)

FDA staining protocol C (Direct-thick-AA) was the same as protocol (B) except that after FDA staining, before being left to dry, an AA decolourisation step was added. For this, AA solution was flooded over the entire slide for 2 minutes and then rinsed with distilled water.

The remaining protocols used thin smears. For thin smears, all FDA staining was done by flooding FDA working solution onto the slide (without filter paper), incubating at 37°C for 30 minutes and then rinsing with distilled water.

#### FDA staining protocol D (Direct-thin-AA)

FDA staining protocol D (Direct-thin-AA) was stained with FDA followed by an AA step.

#### FDA staining protocol E (Direct-thin-AA-phenol)

FDA staining protocol E (Direct-thin-AA-phenol) was same as protocol (D) but after AA for 3 minutes, the slide was flooded with phenol solution for 10 minutes and then rinsed with distilled water.

#### FDA staining protocol F (Direct-thin-AA-phenol-KMnO430s)

FDA staining protocol F (Direct-thin-AA-phenol-KMnO430s) was the same as protocol (E), followed by applying potassium permanganate solution for 30 seconds and then rinsing with distilled water.

#### FDA staining protocol G (Direct-thin-AA-phenol-KMnO460s)

FDA staining protocol G (Direct-thin-AA-phenol-KMnO460s) was the same as protocol (F), except that the potassium permanganate solution was applied for 60 instead of 30 seconds. As shown in Table 1, this protocol has been evaluated previously [12].

#### Conventional acid-fast staining

Smears were flooded with 0.1% auramine for 15 minutes, decolourised with AA for 2 minutes, rinsed with distilled water, flooded with 0.5% potassium permanganate for 30 seconds and then rinsed with distilled water.

#### Culture

**Fig 1:**
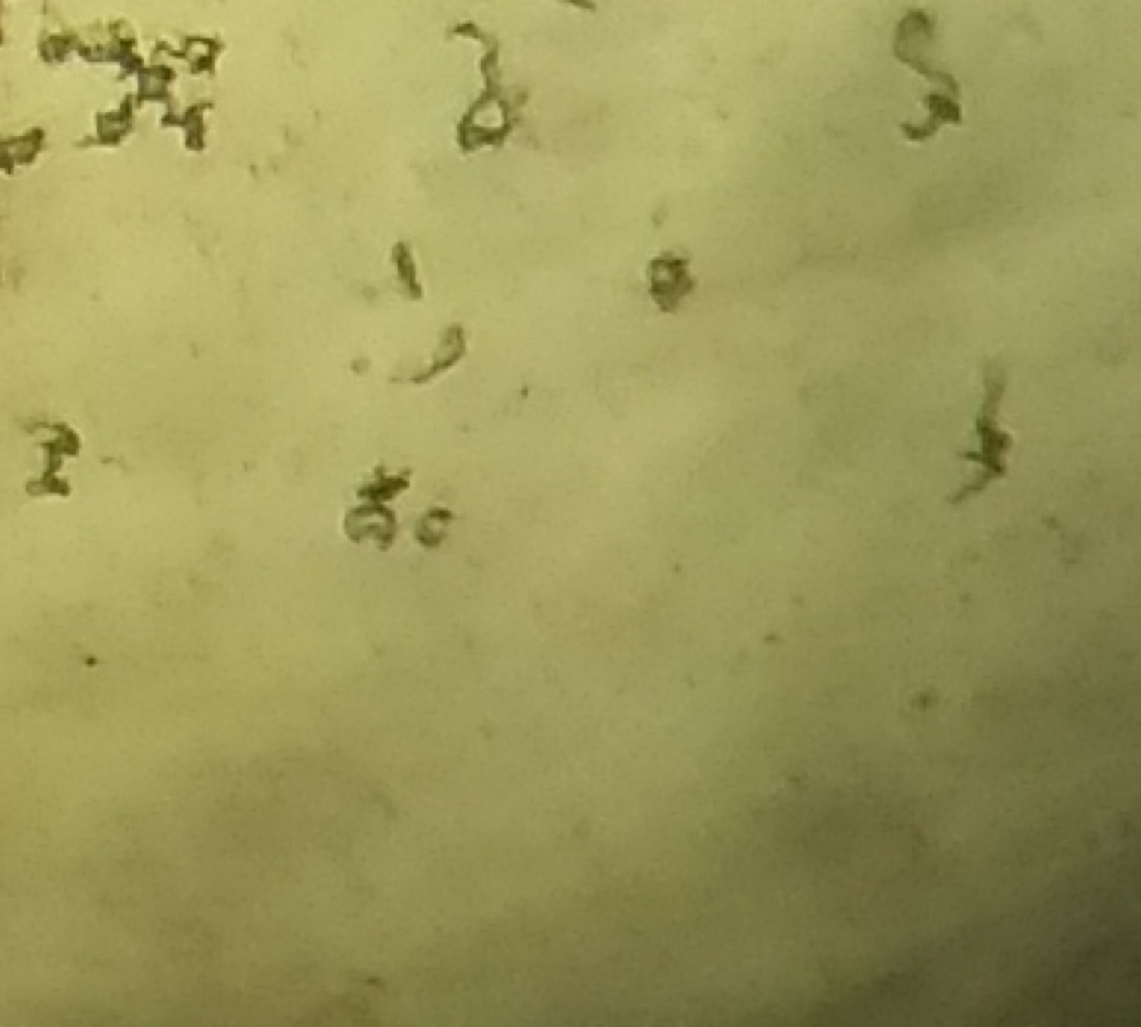
Photograph demonstrating the characteristic cording colony of *Mycobacterium tuberculosis*. There are 11 colonies in this image.

Decontaminated and direct aliquots of sputum samples were inoculated for quantitative culture results using Middlebrook 7H9 culture broth supplemented with glycerol, casitone and the standard oleic acid, albumin, dextrose and catalase (OADC) growth supplement according to the manufacturer’s instructions. To reduce the risk of bacterial or fungal overgrowth contamination, the culture medium was additionally supplemented with Selectatab (Mast Group, Bootle, UK) according to the manufacturer’s instructions, plus 0.25% carbendazim as described [21–23]. The cultures were performed to provide quantitative results in 24-well culture plates, as described [24], preparing a 1:10 dilution by adding 1 drop (approximately 40 μl) of either the unprocessed or decontaminated sputum to a well containing 9 drops (final volume approximately 400 μl) of supplemented Middlebrook 7H9 culture broth. Then 1 drop of this suspension was mixed into another well containing 9 drops of supplemented Middlebrook 7H9 culture broth making a 1:100 dilution and repeated to make 1:1,000, 1:10,000 and 1:100,000 dilutions. Cultures were incubated in un-supplemented air at 37°C. The cultures were sealed in a Ziploc bag, and examined 3-times per week for 6 weeks with an inverted microscope using a 4x objective, final magnification 40x, to detect growth of *M*. *tuberculosis*. Colonies were counted at day 42 of growth, with a colony being defined as a single cell or a clump of cells with the characteristic cording pattern of *M*. *tuberculosis*, as is shown in Fig 1, which is easily distinguishable from other bacteria and filamentous hyphae of fungi. All cultures were done in duplicate.

### Validation study

#### Inclusion criteria

FDA microscopy was introduced into routine laboratory work during 18 November 2015 until January 2016, and applied to all sputum samples from consecutive unselected patients, whether they were about to commence or were already receiving TB treatment, without knowledge of their acid-fast microscopy results.

#### Exclusion criteria

Exclusion criteria were lack of informed written consent or inability to produce a sample.

#### Slide preparation

Three thin smears were prepared from each unprocessed sputum sample.

#### FDA staining

The 2 FDA protocols described above that produced the best results in the optimisation study were re-evaluated in the validation study.

#### Conventional acid-fast staining

For operational reasons, the third thin smear was processed for acid-fast microscopy, as described [20,25], the results of which are not reported here.

### Storage study

#### Inclusion criteria

Sputum was collected from a randomly-selected patient who was already known to their health centre to have strongly sputum smear-positive TB and who had not yet commenced TB treatment, in May 2016. At the same date a negative control sputum sample was collected from a healthy participant with neither symptoms nor suspicion of TB disease.

#### Exclusion criteria

Exclusion criteria were lack of informed written consent or inability to produce a sample.

#### Storage conditions

All sputa and slides were stored protected from light for up to 4 weeks. Direct thin smears were made from each fresh sample and stored at room temperature. The remaining volume of sputa were divided into equal aliquots that were stored at 4°C and at room temperature.

#### FDA staining

Twice a week, direct thin smears were prepared from the patient and the control sputum that were stored at 4°C and at room temperature. Together with patient and control stored slides, these were stained using the optimum FDA protocol, identified in the previous studies described above. All of these procedures were performed in duplicate: 1 slide from each pair of slides was stained using FDA working solution that was prepared daily as described above; the other duplicate from each pair of slides was stained using FDA working solution that had been prepared on the first day of the experiment and stored at room temperature, protected from direct light.

### Cost analysis

All materials and reagents were procured locally in Peru, except for FDA that was obtained in the United Kingdom. An inventory of all reagents was kept and the volume of reagent used for FDA microscopy recorded. With this data, the cost of FDA microscopy using the optimum staining protocol was calculated. Labour costs and equipment such as microscope, glassware, Bunsen burners, drying racks and distilled water were not included in the cost analysis because these costs would have been purchased by a laboratory that was already performing TB fluorescent microscopy with auramine staining, as recommended by the World Health Organization [16].

### Analysis

#### Bacterial counts

The number of stained bacteria visible in 100 consecutive microscopy fields were counted. In culture the number of colony-forming units (CFU) in each serial dilution was recorded. When required, these counts were transformed to the concentration per ml of sample, which was calculated from the volume of sample smeared on the slide or inoculated and diluted for culture [24]. Concentration calculations were used when comparing microscopy results with CFU counts in culture, or microscopy protocols that used different volumes of sputa. When there were duplicate results, geometric means were calculated. Positive microscopy was considered if there was more than 1 bacillus seen per 100 high powered fields in microscopy, and a positive culture defined as more than 1 CFU per well, according to local practice.

#### Quality assessment

If FDA microscopy was positive then during each reading a subjective assessment of quality was made based on the following criteria: background contrast; how easy it was to focus the slide; how bright the bacilli were; and how easy it was to identify bacilli. A score was allocated to each answer, the best being 100 and the worst 0. A total quality score was then calculated as the sum of the individual scores for background, brightness, identification and focus. Therefore, the best score that could be achieved was 400, and the worst was 0.

#### Statistics

As all counts and concentrations were exponentially distributed, results were transformed to their base 10 logarithm (log) for analysis. Because the log of zero values cannot be calculated, before analysis zero values were transformed to the midpoint between zero and the detection threshold. Tests were 2-tailed and were performed with a 95% confidence level and 95% confidence intervals (95% CI). Data with normal distributions were summarised as means (standard deviation, SD) and non-parametric data were summarised as median (inter-quartile range, IQR). When paired data were used to compare results for the same sample processed by different protocols, the paired Student’s t-test was used when the data were normally distributed, and the Wilcoxon signed rank test was used for non-parametric data. Repeated measures were considered for all regression analyses that were used to assess factors that impacted microscopy results and a random effects term was used to adjust for inter-sample variation. When assessing agreement and repeatability between protocols, the limits of agreement method was used, as described by Bland and Altmann [26].

## Results

### Optimisation study

#### Quantitative assessment

There were 9 acid-fast microscopy positive patients and 2 healthy controls who provided samples that were used to make 80 slides and 264 culture wells in this study. All samples from the healthy controls were culture and acid-fast microscopy negative. No cultures failed due to bacterial or fungal overgrowth. Cultures from centrifuge-decontaminated sputa had 5.6% (1/18) false-negative results, whereas directly tested sputa had no false-negative results.

**Fig 2:**
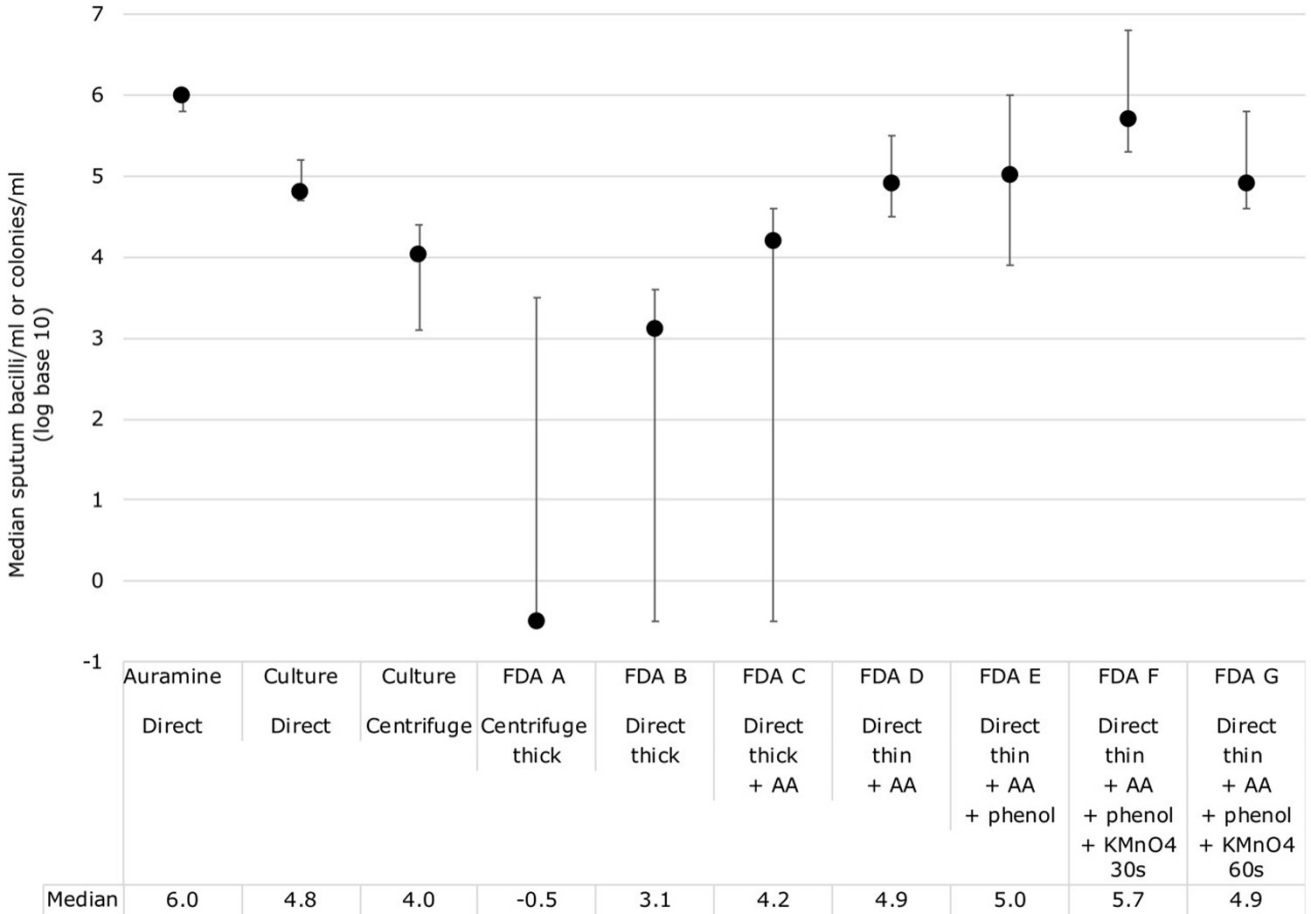
Optimisation study demonstrating the median colony forming units (CFU) or bacilli concentration per ml in each culture and microscopy technique. Error bars indicate the interquartile range. All microscopy and quantitative culture concentration data were transformed to logarithmic (log) base 10 values. Note. FDA = fluorescein diacetate, AA = acid-alcohol, KMnO4 = potassium permanganate, s = seconds.

Fig 2 shows the concentration of CFU/ml in unprocessed sputum culture versus CFU/ml in corresponding decontaminated sputum culture, and bacilli/ml in acid-fast microscopy and FDA microscopy protocols (A-G). Compared to direct sputum culture CFU/ml results, the centrifuge-decontaminated sputum samples had median 10-times less CFU/ml (IQR = 1.8–40 times, P = 0.01). Compared to direct sputum culture CFU/ml results, conventional acid-fast microscopy had a median 12-times (IQR 3.6–63 times) more bacilli/ml (P = 0.01).

**Fig 3:**
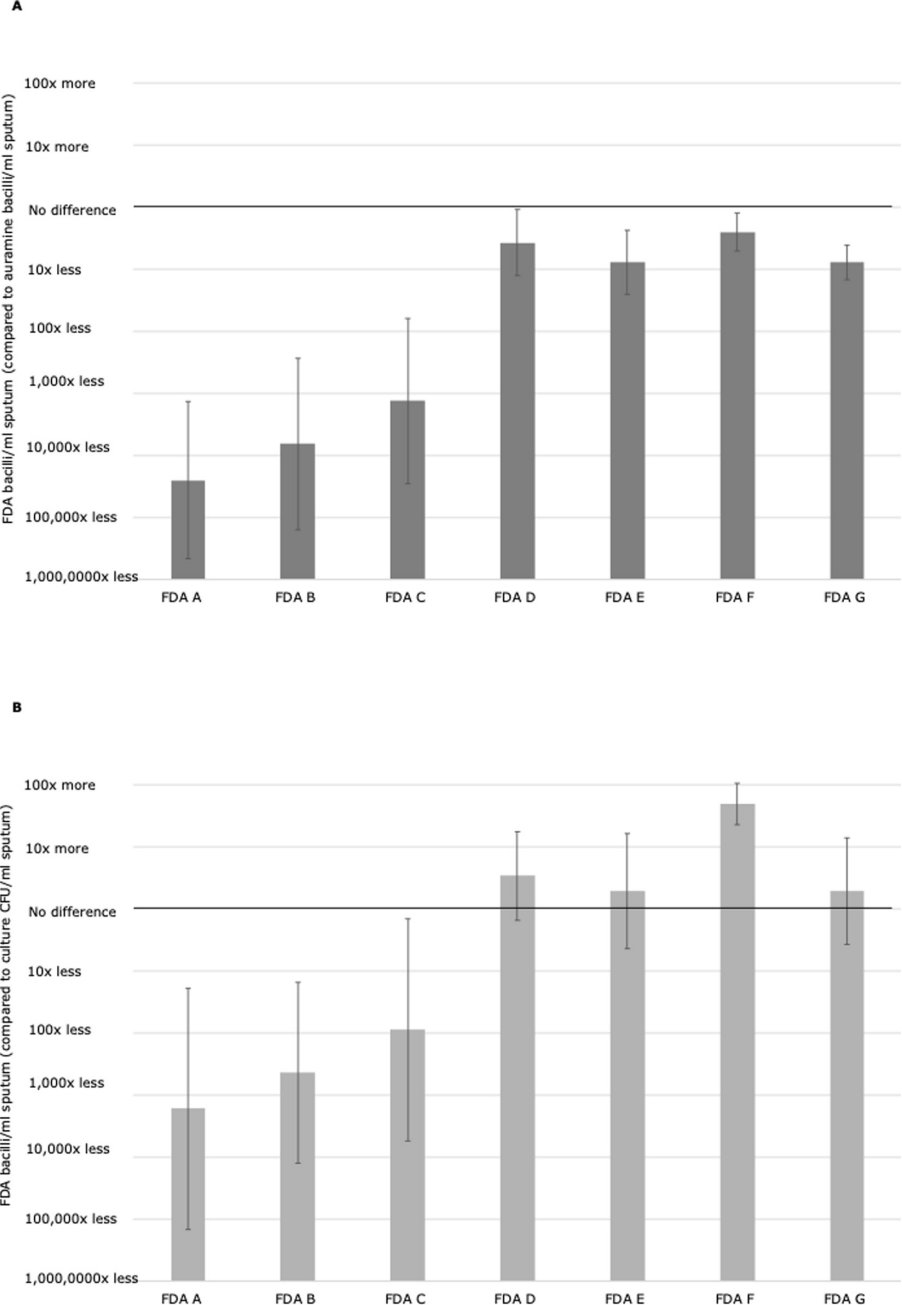
Optimisation study. Bar graphs comparing the quantitative sensitivity of fluorescein diacetate (FDA) microscopy protocols A-G: (A) to identify acid-fast bacilli (AFB), shown as the difference in logarithmic base 10 counts between FDA bacilli/ml of sample versus AFB/ml in acid-fast (auramine) (B) to identify colony-forming units (CFU), shown as the difference in logarithmic base 10 counts between FDA bacilli/ml of sample versus CFU/ml in quantitative culture. Error bars indicate 95% confidence intervals.

In patient samples, 13 slides had false-negative results, of which 11 were stained with protocol A-C. Bacilli/ml results in protocols A-C were lower than other protocols (Fig 2), and were also significantly less than the CFU/ml in the corresponding direct sputum culture (all P≤0.05, Fig 3B). However, protocols D-G had higher bacilli/ml results that had better agreement with the direct sputum culture results (Fig 3B). Specifically, 46% (13/28, 95% CI = 28–66%) of bacilli/ml results in FDA protocols D-G were within +/- 1 logarithm of the corresponding CFU/ml in unprocessed sputa, and 89% (25/28, 95% CI = 72–98%) within +/- 2 logarithms.

Linear regression of the bacilli/ml results for patient samples demonstrated that the factors that predicted higher bacilli/ml in FDA microscopy were: if the protocol used thin (1 drop) versus thick (3 drops) smears (P<0.001); and if the slides were allocated a higher score in the quality assessment (P<0.0001). These 2 variables explained 70% of the within sample variability. The phenol step, which was added for biosafety, did not impact the quantitative results (P = 0.5).

#### Slide quality assessment

Several smears prepared from centrifuge-decontaminated sputum in protocol A were inadvertently partially removed from the slide when the filter paper used during staining was removed. For the protocols using thick smears from direct sputum, identifying bacilli was difficult because they were often obscured by cells and other sputum contents. These factors appeared to contribute to FDA protocols A-C having significantly lower positivity rates in patient samples and lower concentrations of visualised bacilli/ml compared to the CFU/ml in unprocessed sputum culture (Figs 2 and 3, all P≤0.05).

Protocols D, E and G had similar counts (Fig 2), but they differed in quality and therefore ease in reading slides (Table 2). Compared to FDA protocol D, protocol G (which applied potassium permanganate for 60 seconds) had improved background contrast (P = 0.005) and bacilli were easily identifiable. However, slides stained with Protocol G had focusing difficulties and bacilli were less bright (P = 0.05). Therefore, FDA protocol F was later introduced (with 30 seconds of potassium permanganate), which tended to have better background contrast than protocol D (P = 0.08), without any reported focusing difficulties. As shown in Table 2, FDA protocol F and G produced the best quality slides.

### Validation study

#### Quantitative assessment

**Fig 4:**
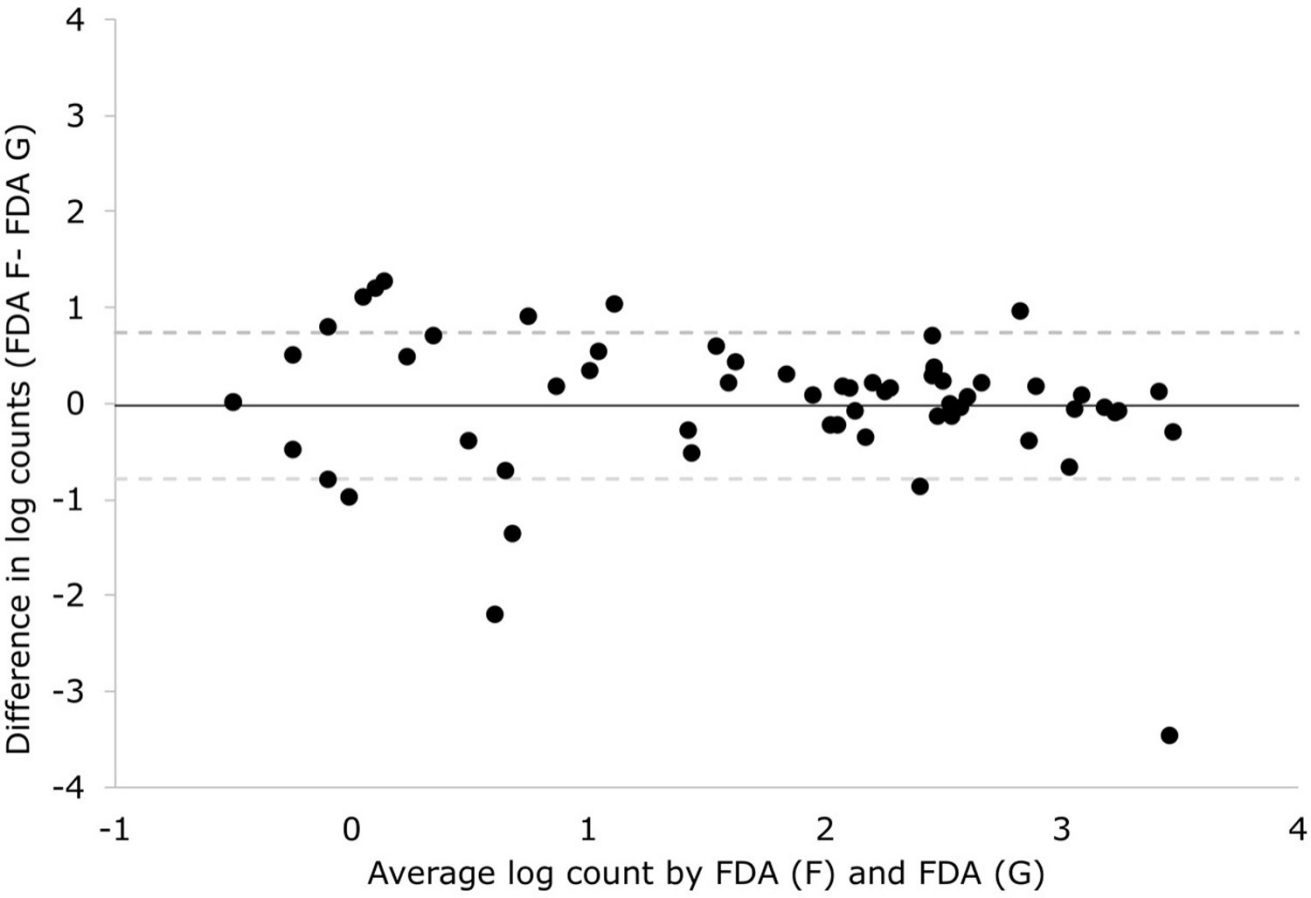
Validation study. A Bland-Altman plot demonstrating agreement between the bacilli count per 100 high powered fields in fluorescein diacetate (FDA) microscopy protocol F versus FDA microscopy protocol G. The difference between these 2 protocols is that the latter involves potassium permanganate quenching for 30 seconds longer. All count data were transformed to their logarithmic base 10 (log) value. The mean difference is -0.007 log (solid line), and the limits of agreement (dotted lines) are -0.70 to 0.71 log.

206 fresh sputa were collected from 200 patients, to prepare 412 slides comparing FDA microscopy protocols F versus G. Sputum was provided by patients with median age 28 years (range = 5–82, IQR = 21–44) and 61% (122/200) were male. Sputum was collected after treatment initiation in 35% (72/206), and 50% (102/206) had a positive conventional acid-fast smear-microscopy result. There were 122 positive FDA microscopy slides, and the number of bacilli visualised by microscopy were similar between the 2 protocols (P = 0.4). Fig 4 demonstrates the high level of agreement between counts in FDA protocol F versus G, with 97% (n/N = 199/206, 95%CI = 93–99%) of results differing by less than +/- 1 logarithm. Fig 4 shows that the agreement between counts tended to be higher in samples with higher bacillary load.

#### Slide quality assessment

Protocol F and G produced similar quality slides (P = 0.3) with median quality score 254 (IQR = 208–292). The time required to count the number of bacilli in 100 high powered microscopy fields was similar for protocol F versus protocol G (P = 0.2) and overall was median 10 minutes (IQR = 9–13). Regression analysis in Table 3 demonstrates that in FDA microscopy-positive samples (N = 122), sputum that did not contain blood had higher quality FDA microscopy slides (odds ratio 6.7, 95% CI = 1.3–34, P = 0.02). However, there were only 6 blood-stained sputum samples in this study (Table 3).

**Table 3:** Validation study. Table demonstrating the factors that improved the quality of slides if fluorescein diacetate (FDA) microscopy was positive (n = 122). The quality score was transformed to a binary variable, above and below the median score, and logistic regression with random effects was used to adjust for inter-sample variation.

		Univariate regression analysis		
Variable		Odds ratio	95% CI	P value
FDA protocol F versus FDA protocol G		1.4	0.57–3.5	0.5
Acid-fast microscopy (grade), ++/+++ % (n)	64% (78)	1.4	0.74–2.7	0.3
Sputum had a salivary consistency, % (n)	42% (51)	0.52	0.15–1.8	0.3
No blood present in sputum, % (n)	90% (110)	13.8	0.97–194	0.05
Delay before processing, median days (IQR)	1 (0–3)	0.93	0.72–1.2	0.6
Delay in reading slide, median hours (IQR)	3.6 (3.1–4.4)	1.5	0.98–2.8	0.2
Rifampicin resistance, % (n)	13% (16)	0.95	0.15–6.0	1.0

### Storage study

#### Quantitative assessment

**Fig 5:**
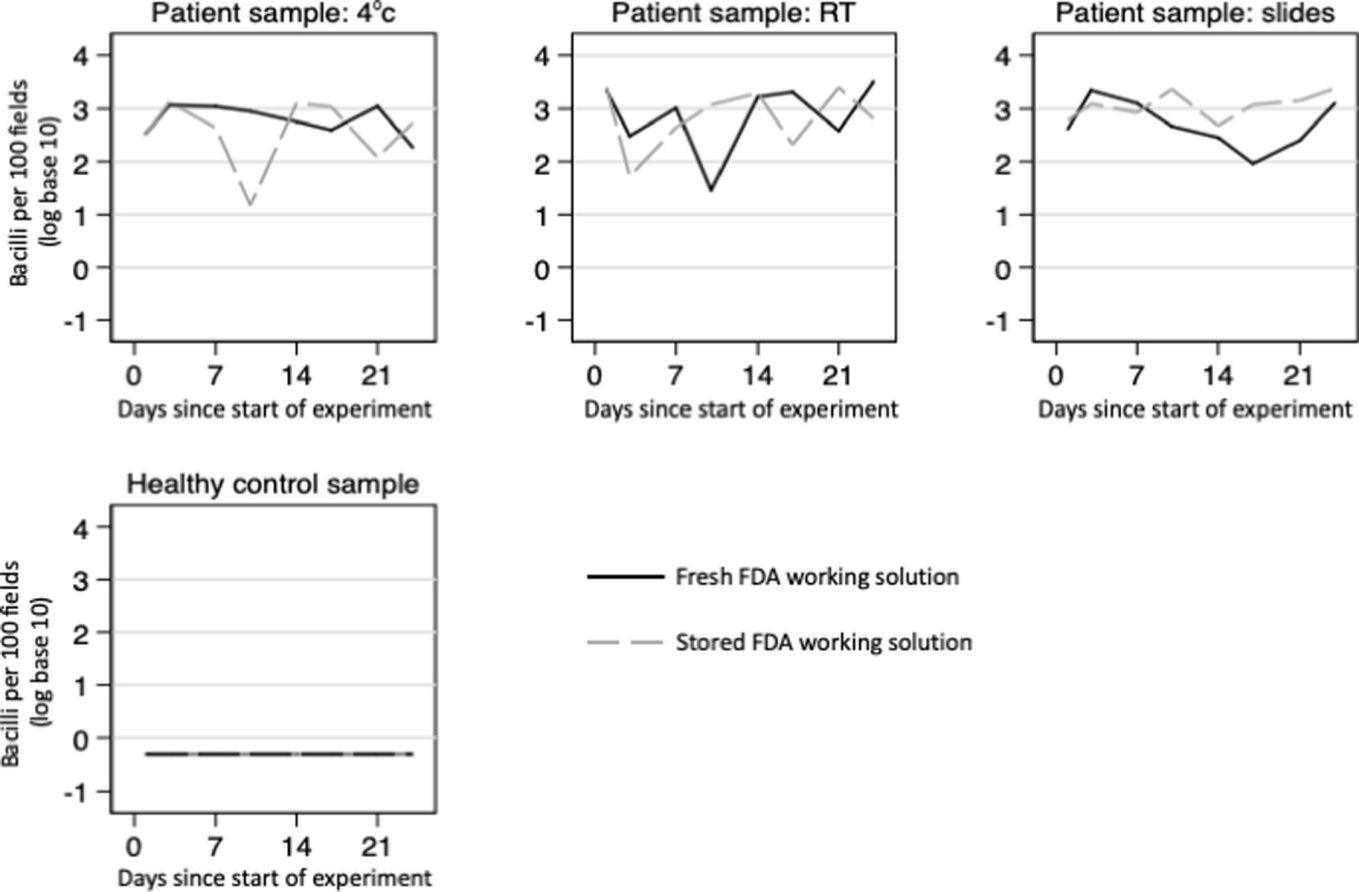
Storage study. Graphs showing the number of bacilli per 100 fields at 8 time-points during the 4-week experiment, comparing fresh versus stored fluorescein diacetate (FDA) working solution in each patient sample storage condition and healthy control sample. Note: RT = room temperature.

For this study FDA protocol F was used to prepare 80 slides: 64 from a patient sputum sample that was conventional acid-fast microscopy +++ smear grade, and 16 from a healthy control. All slides from the healthy negative control participant had negative microscopy results. All slides from the patient with TB had positive results, with FDA microscopy results having a mean log-count per 100 fields of 2.8 (SD = 0.40) on the first day and 2.7 (SD = 0.53) over the next 4 weeks (Fig 5). Neither the way the samples were stored, nor the length of storage impacted the microscopy results (all P>0.2).

#### Quality assessment

The quality of slides for patient samples were high, with median score 300 (IQR = 245–320) out of 400 and this did not change with storage time. Slide quality was more stable if the positive sputum sample was stored on slides and stained with stored FDA working solution than sputum stored at room temperature stained with fresh FDA working solution (P = 0.03).

#### Cost analysis

The material and reagent costs for FDA microscopy using protocol F cost USD $0.02 more than conventional acid-fast microscopy with auramine staining. For a batch of 40 slides, FDA microscopy costs USD $0.05 per slide (Table 4).

**Table 4:** Cost analysis. Table demonstrating the reagents required and costs for fluorescein diacetate (FDA) microscopy using the optimum FDA staining protocol, Protocol F. All reagents except FDA were procured in Peru, and the suppliers are specified in the methods section. Note. USD = United States dollar, mg = milligrams and ml = millilitres.

	Reagent (unit)	Quantity	Price (USD)
FDA stock solution	FDA (mg)	5	$ 0.03
	acetone (ml)	1	$ 0.05
	**Total cost**		**$ 0.08**
FDA working solution for 40 slides	FDA stock solution (ml)	0.1	$ 0.01
	Acetone (ml)	10	$ 0.50
	Phosphate buffered saline (ml)	15	$ 0.01
	**Total cost**		**$ 0.52**
FDA staining for 40 slides	Slide	40	$ 0.69
	FDA working solution (ml)	24	$ 0.52
	0.5% acid alcohol (ml)	24	$ 0.05
	5% phenol (ml)	24	$ 0.19
	0.5% KMn04 (ml)	24	$ 0.02
	Transfer pipette to apply reagents to slide	5	$ 0.55
	**Total cost**		**$ 2.02**
**Total cost per slide**			**$ 0.05**

### Sensitivity and specificity

**Fig 6:**
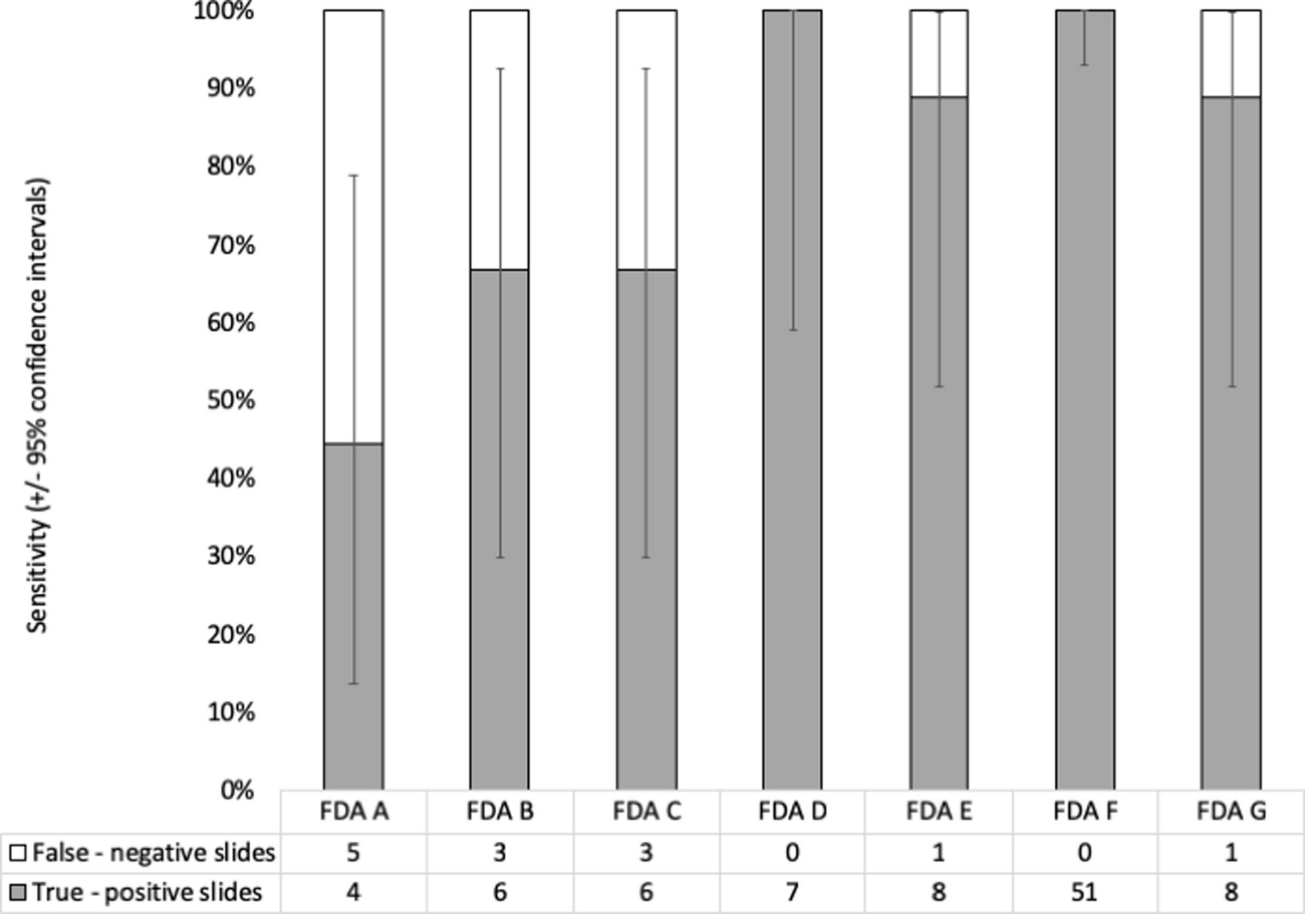
Sensitivity of fluorescein diacetate (FDA) microscopy. FDA sensitivity to detect *Mycobacterium tuberculosis*-positive slides using results from both the optimisation study, which compared FDA microscopy protocols A-G, and the storage study which only used FDA microscopy protocol F. Error bars indicate 95% confidence intervals. Box 1 describes the different FDA microscopy protocols used.

Sensitivity was calculated for slides known to be *M*. *tuberculosis* positive from patients with microbiologically-confirmed tuberculosis prior to treatment initiation, as shown in Fig 6. Analysing the results from both the optimisation study and the storage study, the sensitivity of FDA microscopy protocol F was calculated to be 100% (95% CI = 94–100%). As there were only two negative control samples in the optimisation study, specificity for FDA microscopy protocol F was calculated from slides prepared from negative controls in both the optimisation study (n = 2 slides) and the storage study (n = 18 slides) and was 100% (95% CI = 82–100%).

## Discussion

We compared, optimized, and assessed the reproducibility, optimal logistics and costs of sputum-smear FDA viability microscopy. This enables us to recommend a simple, safe and inexpensive protocol, as shown in the final standard operating procedure (SOP) in Box 2, which we recommend for use in future research and clinical practice. Importantly, this protocol obviates centrifuge-decontamination for FDA microscopy, improving cost, feasibility and biosafety.

**Box 2. Final standard operating procedure (SOP) for fluorescein diacetate (FDA) solution preparation, staining and microscopy.**

**A. Stock solution**

1. Mix 5 mg FDA in 1 ml acetone in a tube.
2. Store at -20°C. Can keep up to 2 years.

**B. Working solution (25 ml)–enough for 40 slides**

1. Put 10 ml of acetone into a clean glass or non-polystyrene tube (because acetone reacts with polystyrene)
2. Add 15 ml of phosphate buffer solution, pH 6.8
3. Cover tube with foil to protect from light
4. Add 0.1 ml of FDA stock solution.
5. Shake very well before use—this is necessary as the FDA stock solution is a suspension and separates within minutes.
6. Store in the dark at room temperature for up to 4 weeks.

**C. Slide cleaning**

1. Remove slides from box
2. Place slides in alcohol for at least 1 hour to clean the slides. We have found this to be particularly important.
3. Removes slides from solution and place on a clean area e.g. a new sheet of aluminium foil.
4. Wipe away excess solution with a clean, lint free cloth or tissue, for example the type of tissue used for cleaning lenses.
5. Do not touch the area where the sample will be applied.

**D. Smear preparation**

1. Use cleaned slides
2. Apply 1 drop of sample to slide, make a smear of approximately 2 cm^2^ area.
3. Leave slides to dry slides. If using a slide warmer, do not use a temperature more than 40°C.
4. Do no expose to ultraviolet light.

**E. Staining protocol**

5. When the slide is dry, pass over flame 3 times to fix the sample to the slide.
6. Shake FDA working solution before use–this is necessary as the FDA stock solution is a suspension and separates within minutes.
7. Apply 13–15 drops of FDA onto samples to cover the smear.
8. Incubate slides for 30 minutes at 37°C.
9. Remove slides from incubator and remove excess liquid by tapping the slide.
10. Rinse GENTLY with distilled water.
11. Apply 0.5% acid-alcohol for 3 minutes.
12. Rinse GENTLY with distilled water.
13. Apply 5% phenol to slide for 10 minutes.
14. Rinse GENTLY and WELL with distilled water. Very important.
15. Apply 0.5% potassium permanganate for 30 seconds.
16. Rinse gently with distilled water.
17. Dry in dark place

**F. Reading protocol**

1. Read slides within 0.5 to 5 hours of staining
2. Use the microscope’s fluorescence light
3. Start with the 40x objective to locate the field of focus
4. Do not move the platform, add a drop of oil, and change the objective to 100x. MAKE SURE NOT TO GET OIL ON THE 40X OBJECTIVE.
5. Now only use the fine focus to focus the slide and start counting the number of bacilli visible with the 100X objective with oil.
6. When the slides are not being read, protect them from light.
7. Start each batch by reading the positive control slide.

**TIPS:**

If focusing is difficult, you could try to focus on the slide label sticker as a reference point and use the fine focus to find the correct plane.

Remember that the more time under light, the less fluorescent the positive bacteria will become because of quenching.

Sputum processing with centrifuge-decontamination is required for most mycobacterial culture methods to reduce cultures becoming unreadable because of overgrowth (often termed contamination) by other non-mycobacterial bacteria and fungi, which are abundant in sputum. Our and other studies have shown that the great majority of culturable *M*. *tuberculosis* in sputum is killed and/or discarded during centrifuge-decontamination [27]. It has also been proposed that sputum processing with centrifuge-decontamination prior to microscopy may increase diagnostic sensitivity by homogenizing sputum and breaking up clumps of *M*. *tuberculosis* [28], but our results do not support this. Centrifugation requires expensive equipment and often the centrifuges that are used with unsealed rotors are biohazardous because they generate infectious aerosols [29,30]. Centrifuge-decontamination may also selectively kill a specific phenotype of *M*. *tuberculosis*, distorting findings important to monitoring treatment response in both quantitative culture and FDA microscopy [23]. It is therefore of considerable operational importance that we found that FDA microscopy had optimal quality and reproducibility when performed directly on unprocessed sputum, without centrifuge-decontamination. Similar to conventional acid-fast microscopy, our proposed FDA protocol uses acid-alcohol to discriminate mycobacteria from non-acid-fast organisms, obviating centrifuge-decontamination.

There is some evidence that conventional sputum smear microscopy is relatively safe for laboratory workers [31]. However, FDA is not toxic to cells, so 5% phenol was applied after staining with FDA to ensure that the slides were sterile because there is abundant evidence that phenolics kill *M*. *tuberculosis* even when dried onto surfaces [32–34]. We found that applying phenol to slides after staining with FDA had no adverse effects on FDA microscopy sensitivity or quality.

We measured the quality of slides in each protocol, because good quality slides facilitate reading and reduce the time required by a microscopist for this process. We found that decontaminated sputum pellets were inadequately fixed onto the standard glass slides that are commonly used in clinical laboratories, resulting in false-negative results. We also found that thick sputum smears produced very poor-quality slides because sputum from patients contained other cells and extra-cellular material that obscured visualization of mycobacteria. Thin sputum smears with the use of 30 seconds of potassium permanganate helped quench fluorescence from background material sufficiently to allow the mycobacteria to be optimally visualized. Consequently, the proposed FDA microscopy protocol required a median 10 minutes to read 100 high-powered fields of patient sputum smear. As microscopists gain more experience this slide reading time may reduce.

Preparing FDA working solution daily is time consuming and potentially wasteful. The storage study established that FDA working solution can be made once every 4 weeks without affecting microscopy results, increasing efficiency. Quality assurance systems are an integral part of providing a reliable TB laboratory service, and have been shown to have a positive impact when implemented [35]. Quality assurance of conventional sputum microscopy is done both internally by the preparation and regular reading of positive and negative controls, and externally, for example by the blinded staining and reading of centrally prepared slides [36,37]. This study demonstrates that quality assurance of FDA microscopy can be carried out in a similar manner, as the storage of positive control slides for up to 4 weeks did not affect results. There was no deterioration in any measures after 4 weeks storage and future research may demonstrate how much longer the reagents and slides may be stored.

The early identification of patients taking inadequate therapy is vital to prevent continued TB transmission in community and institutional settings, and to reduce morbidity and risk of death [13,38–40]. Quantitative results from FDA microscopy can be used to monitor early TB treatment response and predict MDR-TB. The roll-out of TB PCR testing has made settings without culture facilities more frequently able to diagnose rifampicin-resistant TB, although costs including for maintenance and infrastructure are significant barriers to uptake [41]. Furthermore, PCR usually does not identify resistance to other drugs, and cannot differentiate between live and dead *M*. *tuberculosis*, so cannot reliably assess early treatment response [42]. Consequently, even in areas with TB PCR, there may be a role for FDA microscopy in promptly identifying poor response to TB treatment caused by factors other than rifampicin-resistance, and predicting infectiousness [43], which are currently being evaluated in a study in Peru (http://www.isrctn.com/ISRCTN17820976).

FDA microscopy materials were calculated to cost only USD$0.05 per slide, which is USD$0.02 more than for auramine stained conventional acid-fast microscopy. Similar to all microscopy techniques, FDA microscopy cannot guide management in paucibacillary disease. However, this low cost implies that FDA microscopy may be a cost-effective tool to answer clinically relevant questions and formal cost-effectiveness studies are warranted.

Similar to conventional acid-fast microscopy, a limitation of the current FDA microscopy protocol is that it will not be able to differentiate *M*. *tuberculosis* from other acid-fast bacilli, such as non-tuberculous mycobacteria. We did not specifically test the specificity of FDA microscopy on non-mycobacteria microorganisms, but we assume that the acid-fast wash in our protocol would generally prevent them from staining, as is the case for other acid-fast stains. This hypothesis is supported by our finding that all slides prepared from sputum from healthy control participants were consistently negative with the chosen FDA microscopy protocol. Another limitation is that although microscopists were blinded, in the optimization study there may have been clues from the appearance of slides that could have differentiated some protocols (e.g. thick versus thin smears). However, this potential limitation was reduced by taking photos of the microscopy views, which were reviewed by other team members. Finally, the same microscopist read all slides prepared that day; but to remove any systematic bias all FDA slides were shuffled before reading, and the number of bacilli counted in 100 high-powered fields started from the first field in focus without knowledge of the conventional acid-fast microscopy result.

In conclusion, these experiments have optimized and demonstrated the reproducibility of a simple and relatively safe FDA microscopy protocol. This is novel because it provides the first published standard operating procedure recommended for clinical and research laboratories. Furthermore, by demonstrating that centrifuge-decontamination is an unnecessary step, this evidence-based protocol reduces barriers to implementation, especially in resource-constraint settings where FDA microscopy may have most value. This may contribute to TB control efforts and research in areas with the highest prevalence of disease.
